# Addressing clinical uncertainties in ATMP reimbursement: a review of methodological guidelines and European practice

**DOI:** 10.3389/fphar.2026.1749386

**Published:** 2026-03-12

**Authors:** Lotte Delemarre, Isabelle Huys, Walter Van Dyck, Steven Simoens

**Affiliations:** 1 KU Leuven Department of Pharmaceutical and Pharmacological Sciences, Leuven, Belgium; 2 Healthcare Management Centre, Vlerick Business School, Ghent, Belgium

**Keywords:** advanced therapy medicinal products, health technology assessment, indirect treatment comparison, reimbursement, surrogate endpoint

## Abstract

**Introduction:**

Advanced Therapy Medicinal Products (ATMPs) often present substantial clinical uncertainties at the time of reimbursement evaluation, particularly due to the lack of appropriate comparators and the absence of long-term clinical endpoints. This study primarily examined two methodological areas relevant to these challenges: indirect treatment comparisons (ITCs) and surrogate endpoints. In addition, the review was supplemented with an assessment of innovative trial designs to explore how emerging approaches may contribute to evidence generation for ATMPs.

**Methods:**

A structured literature review was conducted using PubMed and Embase, combining keywords such as “Health Technology Assessment,” “ATMP,” “Indirect Treatment Comparison,” and “Surrogate Endpoint,” supplemented with grey literature searches. European pharmacoeconomic guidelines were analyzed using the ISPOR database and referenced national documents.

**Results:**

By April 2025, 27 ATMPs had received European Medicines Agency approval; 20 relied on single-arm trials, and 19 used surrogate endpoints. Single-arm trials limit direct comparative effectiveness assessment, requiring alternative approaches such as ITCs. Conventional ITCs need a common comparator, which is unavailable in single-arm evidence, while population-adjusted indirect comparisons (PAICs) offer a potential solution but depend on strong assumptions and are sensitive to unmeasured confounding. Guidelines generally accept the use of ITCs when direct comparative evidence is unavailable. However, they provide limited guidance on the separate question of when RCTs should be considered infeasible. Surrogate endpoints are widely used due to curative aims of ATMPs, but their validation typically requires RCT evidence and is highly context-specific. Guideline acceptance criteria for surrogate endpoints vary. Some require robust validation demonstrating a causal relationship, while others allow correlation-based or context-specific justification, particularly when direct evidence is limited or the technology addresses rare or serious conditions. Innovative trial designs may help generate more robust evidence in these challenging contexts.

**Discussion:**

The study highlights the need to refine methods for handling single-arm trials, projecting long-term outcomes, and using surrogate endpoints in ATMP evaluations. While some clinical uncertainty is inevitable, healthcare systems must manage it effectively to ensure timely, equitable access without creating additional barriers.

## 1 Introduction

Advanced Therapy Medicinal Products (ATMPs), as defined by the European Medicines Agency (EMA), are medicinal products based on genes, tissues, or cells (European Parliament and Council of the European Union, 2007). This category encompasses gene therapies, somatic-cell therapies, tissue-engineered medicines, and advanced therapies combined with medical devices. As of April 2025, 27 ATMPs had received marketing authorisation within the European Union (European Medicines Agency, 2025).

ATMPs have prompted a significant shift in pharmaceutical development by introducing treatments that often aim to cure (Detela and Lodge, 2019). Conventional medicines are typically designed to alleviate symptoms and slow further progression, often requiring continuous administration over a patient’s lifetime. In contrast, many ATMPs can achieve lasting therapeutic effects with a single dose (Vestergaard et al., 2013). They are also frequently tailored to individual patients, moving beyond the standardized “one-size-fits-many” model of traditional drug development (Ledford, 2025).

These distinctive features present substantial challenges for the clinical evaluation of ATMPs. A prominent challenge is the frequent absence of a relevant comparator (Pinho-Gomes and Cairns, 2022). ATMPs are often developed for life-threatening diseases currently lacking effective treatments, with only best supportive care available. For these conditions, where no other therapeutic options exist, it can be considered unethical to withhold a potentially life-saving ATMP from patients by assigning them to a placebo or an inferior comparator arm in a clinical trial (Cargill et al., 2021). Furthermore, many ATMPs hold curative potential, meaning their full clinical benefit may only manifest years or even decades post-treatment. These challenges are further complicated by the fact that ATMPs often target rare diseases with very small patient populations (Hanna et al., 2016). This inherent rarity severely limits the feasibility of conducting robust comparative studies, thus contributing to considerable clinical uncertainties.

These challenges in the clinical evaluation pose considerable difficulties for subsequent reimbursement decisions (Iglesias-Lopez et al., 2021a). Health Technology Assessment (HTA) bodies typically assess the added therapeutic value of new interventions by comparing them to existing standards of care. This evaluation predominantly relies on comparative effectiveness data, often derived from RCTs (Fontrier et al., 2022). The absence of direct comparators for many ATMPs, coupled with the reliance on small, often single-arm studies, complicates the quantification of the incremental health benefit in a manner consistent with established HTA methodologies (Gozzo et al., 2021). Another key challenge is the frequent lack of long-term data on the durability of clinical outcomes. This uncertainty limits the ability to fully assess their long-term value within the typical timeframes used in HTA. A possible solution for this is the use of surrogate endpoints, which can provide earlier indications of a therapy’s potential long-term effectiveness (Gladwell et al., 2024). While these therapies often entail high upfront costs, their potential long-term health benefits and cost offsets remain unproven at the time of evaluation.

To our knowledge, two articles to date have provided an overview of the clinical uncertainties associated with ATMPs alongside potential methodological solutions (Iglesias-Lopez et al., 2021b; Qiu et al., 2022). In this article, we aim to delve deeper into the methodological strategies that can address these uncertainties and explore how various national pharmacoeconomic guidelines within Europe have responded to them. We specifically focus on two major challenges: the frequent absence of a relevant comparator and the lack of long-term clinical endpoints at the time of evaluation. These topics are especially important as an increasing number of single-arm trials, ITCs, and studies using surrogate endpoints are being submitted to reimbursement agencies (Patel et al., 2021; Harricharan et al., 2021; Grigore et al., 2020; Wheaton and Bujkiewicz, 2025). Methodologies for managing small patient populations, while relevant, were not a primary focus of this analysis, given the recent extensive literature on the topic (Day et al., 2018; Friede et al., 2018).

## 2 Methods

### 2.1 Review design

A narrative, structured literature review was conducted between November 2024 and February 2025. The objective was to identify all relevant evidence on clinical uncertainties in ATMPs, with a particular focus on the use and assessment of indirect treatment comparisons (ITCs) and surrogate endpoints within HTA processes.

### 2.2 Search strategy and information sources

A predefined search strategy was implemented in PubMed and Embase, combining keywords and controlled vocabulary terms related to: Health Technology Assessment, Advanced Therapy Medicinal Products, Indirect Treatment Comparison, and Surrogate Endpoints. The search strategy was developed with the support of a medical librarian to ensure comprehensive coverage of terminology and databases.

The initial search strategy was intentionally broad to capture all relevant methodological discussions in HTA. During title and abstract screening, publications were retained if they explicitly referred to ATMPs or addressed HTA methodological issues relevant to ATMP assessment.

The search was supplemented with grey literature retrieval, including HTA bodies’ guidelines, reports from international organizations, and documents issued by relevant professional bodies.

To enhance completeness, a snowballing approach was applied by screening the reference lists of all included publications and relevant reviews.

### 2.3 Eligibility criteria

Eligible publications were peer-reviewed articles written in English and reporting findings from literature reviews or document analyses. Authoritative grey literature from recognized institutional sources was also included. Clinical studies and non-peer-reviewed documents outside the defined grey literature scope were excluded.

### 2.4 Study selection

**FIGURE 1:**
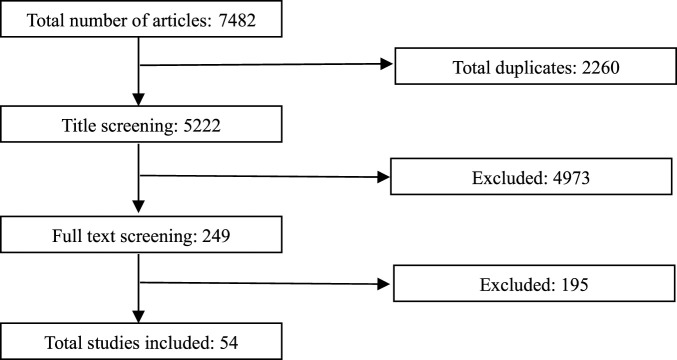
Flowchart illustrating the study selection process.

One reviewer screened titles and abstracts followed by full-text assessment. The screening and selection process is summarized in the flowchart (Figure 1). Any methodological approaches outside the primary focus were categorized under innovative trial design if identified during the search.

During title and abstract screening, publications were retained if they referred to ATMPs or to HTA methodological challenges plausibly applicable to ATMP assessments. During full-text screening, inclusion was restricted to articles explicitly addressing how HTA bodies cope with clinical uncertainties related to surrogate endpoints and indirect treatment comparisons in the evaluation of ATMPs.

### 2.5 Comparative analysis of pharmaco-economic guidelines

For the comparative assessment of pharmacoeconomic recommendations, we focused on European countries. This geographical scope aligned with the evaluation of clinical trial evidence available at the time of EMA marketing authorisation, ensuring a consistent regulatory context. Relevant documents were identified using the ISPOR database on pharmacoeconomic guidelines, supplemented by national HTA guidance from selected European countries (ISPOR, 2025).

### 2.6 Data extraction and synthesis

Data from the included publications were charted according to relevance to clinical uncertainties in ATMPs, with particular attention to indirect treatment comparisons, surrogate endpoints, and innovative trial designs where applicable. Results were synthesized narratively due to heterogeneity in study types, evidence sources, and reporting formats.

## 3 Results

### 3.1 Indirect treatment comparison

This section discusses and explores approaches to address clinical uncertainty surrounding the relative effectiveness measurement of an ATMP versus a comparator in a reimbursement context.

#### 3.1.1 RCT as the gold standard

RCTs are considered as the gold standard to measure treatment outcomes. Randomization secures comparability of participants characteristics in both treatment and control or comparator group. This reduces bias and allows to examine the cause-effect relationship between an intervention and an outcome. This principle applies not only to known or observed patient characteristics but also to unknown characteristics, where achieving or even assessing a balanced distribution across groups is not feasible using alternative methods (Collins et al., 2020).

For some ATMPs evidence from RCTs is available (e.g., Luxturna®) (Russell et al., 2017). However RCTs are not always feasible, due to practical or ethical constraints. From an ethical perspective, ATMPs are often developed for life-threatening diseases with limited or no existing treatment options. Requiring patients to participate in RCTs, where some may receive suboptimal standard of care instead of an effective treatment option, raises ethical concerns (Frangoul et al., 2024). For some therapies, no commonly accepted standard of care exists, complicating the choice of comparator (Talib and Boon, 2020). Small patient populations and the complex manufacturing of these therapies further limit trial feasibility (Hanna et al., 2016). In therapies like CAR-T cell treatment, multiple relevant comparators make comprehensive RCTs impractical and resource-intensive.

However, there are currently no clear guidelines for determining the feasibility of conducting an RCT for ATMPs. There is a need for clear guidance to ensure that feasibility assessments are carried out collaboratively with stakeholders at an early stage. If an RCT is deemed not feasible, alternative methods for generating comparative evidence with single-arm trials, such as indirect treatment comparisons, should be considered (Julian et al., 2022).

#### 3.1.2 Characteristics of indirect treatment comparisons

The term indirect treatment comparison (ITC) is mostly used to describe the comparison of the effectiveness of two or more treatments that have not been directly studied in head-to-head trials and rely on a common comparator. However the term can also be used to refer to any study type in which treatment groups from different studies are compared. When RCTs are unavailable and indirect treatment comparisons are used, it is important to recognize that this approach inherently increases uncertainty about the treatment effect, as it cannot ensure the same level of balance for both known and unknown effect modifiers. Therefore, direct comparisons in RCTs with a low risk of bias should be used whenever possible (European Commission, 2024a). Numerous guidelines on ITCs have been issued by regulatory and other authorities such as the Canadian Agency for Drugs and Technologies in Health (Canadian Agency for Drugs and Technologies in Health, 2009), the Food and Drug Administration (U.S. Food and Drug Administration, Center for Drug Evaluation and Research, 2023) and the EU Member State Coordination Group on HTA (European Commission, 2024a), but the main methods for performing an ITC are summarized below including their features, assumptions, (dis)advantages, and the specific circumstances in which they are most appropriate.

All ITC methods rely on the assumption of exchangeability, meaning that substituting patients from one trial with those from another would not change the expected treatment effect. This is assessed through homogeneity, similarity, and consistency (Macabeo et al., 2024a).

- Homogeneity: No variation in relative effectiveness between treatment pairs, ensured by comparable trial designs, patient populations, interventions, and outcomes.
- Similarity: Trials are sufficiently alike regarding effect modifiers, so differences in known or unknown factors that influence outcomes are minimal.
- Consistency: No meaningful differences exist between direct and indirect evidence; significant discrepancies or reversed treatment effects raise concerns.

A number of methods can be used to carry out an ITC (U.S. Food and Drug Administration, Center for Drug Evaluation and Research, 2023; Macabeo et al., 2024a; Tanaka et al., 2024; European Commission, 2024b; Ades et al., 2024).

##### 3.1.2.1 Standard methods

Standard ITC methods, including the Bucher method and network meta-analysis (NMA), enable the estimation of relative treatment effects in the absence of direct head-to-head trials. The Bucher method facilitates indirect comparisons within a simple three-treatment network linked by a common comparator, whereas NMA integrates both direct and indirect evidence across more complex networks, potentially including multiple interventions. Both approaches require the assumptions of homogeneity, similarity, and, in the case of NMA, consistency. These methods can provide robust comparative effectiveness evidence when multiple comparators exist, as exemplified by CAR-T therapies, but they cannot overcome the inherent limitations of single-arm trials.

##### 3.1.2.2 Population-adjusted methods

Population-adjusted ITC (PAICs) methods are used to adjust for imbalances in treatment effect modifiers between studies (Phillippo et al., 2018). Anchored methods rely on a common comparator connecting two or more trials, allowing relative treatment effects to be estimated while preserving the randomised design of the original studies. In contrast, unanchored methods are applied when no common comparator exists, such as in single-arm trials, and estimate treatment effects without any shared reference. Although unanchored ITCs can provide comparative effectiveness evidence in settings where only single-arm data are available, they remain susceptible to bias from unknown or unmeasured effect modifiers, which cannot be accounted for (Hatswell and Sullivan, 2020; Thom et al., 2022).

Propensity score methods use individual patient data (IPD) from both studies to balance measured covariates across treatment groups, thereby reducing confounding. When IPD are available for only one treatment and only aggregate data (AGD) exist for the comparator, Matching-Adjusted Indirect Comparison (MAIC) and Simulated Treatment Comparison (STC) can be applied. MAIC weights patients in the IPD trial to match the baseline characteristics of the aggregate population, while STC uses regression models on the IPD to adjust outcomes.

Consequently, transparent reporting of the rationale for using a PAIC, the selection and clinical justification of treatment-effect modifiers, data sources, balance diagnostics, and sensitivity analyses exploring residual confounding is essential for the interpretation of these analyses in HTA (Phillippo et al., 2018; Signorovitch et al., 2012).

#### 3.1.3 The role of indirect treatment comparison for ATMPs

As of April 2025, 27 ATMPs have obtained market authorisation from the EMA (European Medicines Agency, 2025). Of these, only seven were supported by RCTs, whereas 20 relied on single-arm evidence. This evidentiary landscape highlights the increasing reliance on ITCs to inform comparative effectiveness assessments in ATMP evaluations (Table 1).

**TABLE 1:** Key characteristics of ATMP studies.

ATMP	Type of ATMP	Non-randomised	Historical control	Surrogate endpoint
Chondrocelect	TEP	​	​	X
Glybera	GTMP	X	​	X
MACI	TEP	​	​	X
Provenge	CTMP	​	​	​
Holoclar	TEP	X	​	X
Imlygic	GTMP	​	​	X
Strimvelis	GTMP	X	X	​
Zalmoxis	CTMP	X	X	X
Spherox	TEP	​	​	X
Alofisel	CTMP	​	​	​
Yescarta	GTMP	X	X	X
Kymriah	GTMP	X	X	X
Luxturna	GTMP	​	​	X
Zynteglo	GTMP	X	​	X
Zolgensma	GTMP	X	X	​
Libmeldy	GTMP	X	X	X
Tecartus	GTMP	X	​	X
Skysona	GTMP	X	X	​
Abecma	GTMP	X	X	X
Breyanzi	GTMP	X	​	X
Carvykti	GTMP	X	​	X
Upstaza	GTMP	X	X	​
Roctavian	GTMP	X	​	X
Ebvallo	CTMP	X	​	X
Hemgenix	GTMP	X	​	​
Casgevy	GTMP	X	​	X
Durveqtix	GTMP	X	​	​

HTA agencies continue to express caution regarding ITCs, particularly with respect to population-adjusted approaches and most prominently unanchored methods, as these analyses are not randomised and therefore depend on strong, often unverifiable assumptions that can introduce bias. Despite this hesitancy, unanchored population-adjusted ITCs may represent the only viable option when comparative effectiveness from a single-arm trial with historical or external comparators are required. However, their adoption has gained traction because naive comparisons constitute the sole alternative in such contexts (Macabeo et al., 2024b; Katsoulis et al., 2024).

Unanchored population-adjusted ITCs require robust adjustment for all imbalanced prognostic factors and treatment-effect modifiers. In practice, satisfying the assumption of no unobserved confounding is widely considered challenging or even infeasible. The difficulty is amplified by the fact that reporting practices remain highly heterogeneous and frequently incomplete (Truong et al., 2023; Serret-Larmande et al., 2023). Accurate selection and transparent reporting of relevant confounders are crucial. Without these, adjusted estimates may not demonstrate improved validity or precision compared with unadjusted analyses. This situation illustrates the need for strengthened methodological and reporting standards, and recent initiatives have begun to formulate recommendations to address these shortcomings (Freitag et al., 2023).

In addition, population-adjusted ITCs in the ATMP field are conducted almost exclusively in collaboration with pharmaceutical manufacturers. In such analyses, the IPD arm typically represents the sponsor’s own therapy, while the aggregated data correspond to a competitor’s treatment. Ideally, appropriate adjustment would require IPD for both interventions (Phillippo et al., 2018; Bachy, 2024). However, such data are rarely available. Historically, comparator evidence has mainly consisted of clinical trial control groups. Real-world external controls are increasingly considered preferable, as they enable the construction of contemporary standard-of-care cohorts that better reflect current clinical practice.

Several PAICs have been conducted in Spinal Muscular Atrophy to compare ATMPs against therapies such as nusinersen and risdiplam (Bischof et al., 2021; Ribero et al., 2022). However, differences in trial populations, baseline characteristics, and outcome definitions, along with limited covariate adjustment, leave substantial residual confounding and reduce the validity of these indirect comparisons (Jiang et al., 2023). Similarly, CAR-T therapies are often used in late-line relapsed or refractory settings, and the presence of multiple approved CAR-T products for the same indications further complicates the feasibility of RCTs. Network meta-analyses and PAICs have been performed to compare outcomes across treatments in B-cell non-Hodgkin lymphoma and multiple myeloma (Looka et al., 2025; Liao et al., 2024; Shah et al., 2022; Jagannath et al., 2021; Alsdorf et al., 2024), but prior studies frequently failed to account for key clinical factors, such as prior treatment history and treatment duration, leading to potentially biased or clinically limited estimates (Cope et al., 2020).

#### 3.1.4 A comparative analysis of pharmacoeconomic guidelines in Europe regarding indirect treatment comparisons

Hence, for this study, we reviewed pharmacoeconomic guidelines from various European countries that are available in English (see Table 2). Nearly all guidelines strongly emphasize a preference for RCTs while acknowledging the use of indirect treatment comparisons. The only justification given for ITC is the lack of direct comparisons, without specific guidelines on when ITC should be considered appropriate. Nearly all guidelines agree that unadjusted indirect comparisons are inappropriate. Within adjusted ITCs, network meta-analysis is most often mentioned for comparing placebo-controlled trials. However, ATMP evaluations often lack placebo-controlled trials and instead rely on single-arm trials.

**TABLE 2:** HTA guidance on the use and preference for ITC methods.

Country	Standard methods	Population-adjusted methods
The Netherlands	NMA or MTC preferred method of ITC	A single-arm study alone cannot determine relative effectiveness. Therefore, it must be combined with an external control arm for indirect comparison, which introduces significant uncertainty and is never the preferred approach
Denmark	If direct comparison data is unavailable, the company should conduct indirect comparisons using pairwise-adjusted methods, NMA, or other validated approaches	The company should use statistical methods to enhance comparability, adjusting relevant parameters. MAIC or STC can be applied
Belgium	If no direct comparisons are available, indirect comparisons from RCTs can be performed	/
France	The chosen evaluation method should be justified and clearly detailed, an NMA is recommended	A population adjusted indirect comparison method may be used if justified
Germany	Only adjusted indirect comparisons using appropriate common comparators are accepted, including the Bucher method and NMA	MAIC and STC are disapproved due to strong assumptions about unknown effects and reliance on non-testable causal models without a common comparator
Ireland	Adjusted indirect comparison is suitable for two technologies with a common comparator	Single-arm study data should be included only with an anchored comparison
Norway	In the absence of direct efficacy data between the intervention and comparators, indirect comparisons can be performed, including pairwise adjusted indirect comparisons, NMA, or other validated methods	If individual patient data is available for at least one study, methods such as MAIC or STC can be used, provided the relevant conditions for these methods are met
Switzerland	If no direct head-to-head studies are available for two comparators, indirect comparisons can be made using individual studies. The limitations of indirect comparisons due to study differences must be thoroughly discussed	/
Finland	If health effects are assessed through indirect comparisons, proper scientific practices must be followed in both their preparation and reporting	/
Scotland	Elaborate guidelines with a checklist of information for indirect evidence	/
England	When technologies are being compared that have not been evaluated within a single RCT, data from a series of pairwise head-to-head RCTs should be presented together with an NMA if appropriate	Population adjustment methods in connected networks can be used when effect modifiers are imbalanced between trials. The limitations of these methods should be acknowledged, along with an estimate of any potential systematic bias
Portugal	If the evidence base consists only of randomised studies but has disconnected elements, alternative methods can link them. Preferably, this includes expanding the network to incorporate 2nd and 3rd order indirectness	The least preferred approach is the use of unanchored adjustment methods (MAIC, STC) or observational data, as these analyses require careful interpretation

The German guidelines are particularly stringent, explicitly disapproving of MAIC and STC due to the strong assumptions required about unknown effects (Institute for Quality and Efficiency in Health Care (IQWiG), 2022). Similarly, though less stringent, the Dutch guidelines state that such methods imply significant uncertainty and are therefore never the preferred approach (Zorginstituut Nederland, 2024). The Portuguese guidelines rank ITC methods hierarchically, placing MAIC and STC at the lowest tier and advising that results from these analyses should be interpreted with caution (INFARMED ‐ National Authority of Medicines and Health Product, 2019).

Other guidelines, such as those from France, Norway, Denmark and Scotland, provide more permissive perspectives. They indicate that population adjusted indirect treatment methods can be considered provided that robust statistical methodologies are employed. Moreover, these guidelines emphasize the need to justify the chosen method, and biases must be discussed and explained.

Overall, there is a pressing need for more comprehensive guidelines addressing single-arm trials, particularly when systematic searches reveal an absence of suitable comparators. The Scottish guidelines stand out by offering clear guidance on the use of indirect treatment comparisons, setting a potential model for addressing these challenges in ATMP reimbursement evaluations.

### 3.2 Surrogate endpoints

This section discusses and explores approaches to address the use of surrogate endpoints in the assessment of ATMPs for reimbursement purposes.

A clinical endpoint, also referred to as a patient-relevant outcome, is a direct measure of how a patient feels, functions or survives (Biomarkers, Definitions Working Group, 2001). Surrogate endpoints are used to replace and predict a clinical endpoint that cannot be observed in the trial. They can be a biomarker or an intermediate clinical endpoint (Ciani et al., 2017). Whilst there is a preference for clinical endpoints, the need to use surrogate endpoints is recognised (Ciani et al., 2023). Surrogate endpoints are increasingly used in clinical research because they allow earlier assessment of treatment efficacy when long-term outcomes require extended follow-up (Ciani et al., 2021). They are particularly valuable for diseases with small patient populations, as surrogate endpoints can be measured more frequently, allowing trials to demonstrate treatment effects with fewer participants. In severe or life-threatening conditions, their use can also be ethically justified, since waiting for long-term clinical outcomes may place patients at unnecessary risk. EUnetHTA has developed guidelines on the use of surrogate endpoints, which have been adopted by international HTA bodies (Grigore et al., 2020). These guidelines state that the acceptability of a surrogate endpoint is based on biological plausibility and empirical evidence (EUnetHTA, 2015). There are three levels of evidence taken into account for the endpoint-clinical outcome relationship (Fleming and DeMets, 1996). Level one evidence is established by assessing the correlation coefficient between the treatment effect on the surrogate endpoint and the clinical endpoint across multiple RCTs or through meta-analysis. Level two evidence is established by demonstrating a consistent association between the surrogate endpoint and the clinical endpoint from epidemiological/observational studies. Level three evidence is based solely on biological plausibility, derived from pathophysiological studies or an understanding of the disease process. Furthermore, the validation of surrogate endpoints appears to be disease specific, population specific and technology specific.

The use of surrogate endpoints introduces uncertainty into the assessment of clinical benefit, which can lead to differing incentives for regulators and payers. Regulators may approve therapies to enable early patient access, while payers face uncertainty about true clinical outcomes and adopt more cautious coverage decisions (Dawoud et al., 2021; Bognar et al., 2017). HTA bodies prefer that not only the primary endpoint, but also all other relevant endpoints are clinical endpoints (Pavlovic et al., 2014). Surrogate endpoints are generally considered only when clinical endpoints are unavailable and the surrogate endpoint is validated (Velasco Garrido and Mangiapane, 2009). However, a recent study found that the use of surrogate endpoints was not associated with an increased likelihood of a negative reimbursement decision in cancer drugs (Pinto et al., 2020). In line with this, an analysis of HTA reports and a choice experiment study revealed that HTA bodies’ acceptance of surrogate endpoints does not necessarily translate into a greater likelihood of positive coverage decisions (Ciani et al., 2022). In contrast, cost-effectiveness emerged as a significant determinant in these decisions, consistent with findings from earlier research (Ledford, 2025). However, cost-effectiveness analyses frequently involve extrapolating surrogate endpoints to predict clinical outcomes (Taylor and Elston, 2009). Previous systematic reviews have demonstrated that drugs approved on the basis of surrogate endpoints often fail to show a corresponding benefit on definitive clinical endpoints (Wheaton and Bujkiewicz, 2025; Cooper et al., 2020).

#### 3.2.1 The role of surrogate endpoints for ATMPs

As of April 2025, a total of 27 ATMPs have been approved by the European Medicines Agency (European Medicines Agency, 2025). Of these 19 relied on the use of surrogate endpoints (see Table 1).

Due to their curative potential and intended long-term clinical benefits, ATMPs significantly rely on surrogate endpoints to demonstrate efficacy for reimbursement when definitive outcomes, like overall survival, have not yet matured (Ciani et al., 2023). However in the context of ATMPs, it is challenging to meet the prerequisites for the validation of surrogate endpoints as described above (Gladwell et al., 2024). Level one evidence requires multiple RCTs that demonstrate a robust trial-level association between treatment-induced changes in the surrogate endpoint and corresponding changes in the ultimate outcome of interest. Moreover, such trials must be conducted in the same target population, for the same disease, and within the same therapeutic class as the novel intervention. As noted above, ATMPs are a novel technology often developed for diseases with no existing treatment options. Consequently, RCT data are frequently unavailable, and surrogate endpoints validated in other contexts cannot be directly transferred, as their applicability is technology-, disease-, and population-specific. To help address these challenges, new methods are developed such as the causal inference approach with mixed models for longitudinal outcomes, to establish robust validation using limited single-trial data (Roberts et al., 2023). Another example of such a novel methodology is the use of Bayesian Evidence Synthesis methods to formally integrate real-world evidence with trial data for a more robust surrogate endpoint validation (Wheaton et al., 2023). For oncology, there is a substantial literature on the relationship between progression-free survival and overall survival, but this discussion is beyond the scope of the current literature review. A number of factors affect this relationship and should consider both tumour-type and other relevant clinical and patient factors (Assouline et al., 2022; Sola-Morales et al., 2019; Lux et al., 2021; Campbell et al., 2019; Holstein et al., 2019).

In addition to the reliance on surrogate endpoints, HTA bodies are also required to extrapolate clinical data to estimate long-term outcomes such as overall survival and sustained clinical benefit, which are rarely fully observed within the limited duration of clinical trials (Hardy and Hughes, 2022). This extrapolation is particularly important for cost-effectiveness analyses, as projected lifetime health gains strongly influence value assessments and reimbursement decisions. Multiple modelling techniques exist to extrapolate clinical data, with techniques having different characteristics and making different assumptions (Latimer, 2013). Nevertheless, the assumptions underpinning extrapolation models introduce significant uncertainty, and even small variations in survival curve modelling can lead to markedly different incremental cost-effectiveness ratios (Tai et al., 2021). One proposed approach to managing this persistent uncertainty is to embed reassessment into the HTA and reimbursement process. Reassessing a technology at predefined intervals aligns well with the long-term evidence generation pathways of ATMPs, enabling decisions to be updated as more mature data become available. Such a life-cycle approach would ensure that products with uncertain durability of benefit are not prematurely locked into reimbursement conditions (Versteeg et al., 2024). To mitigate these challenges, it would be helpful if reimbursement agencies clarify which factors they take into account when judging the maturity of clinical data and to offer guidance on validated modelling practices. Extensive sensitivity analyses, and the integration of real-world evidence are increasingly emphasized in HTA evaluations of ATMPs.

#### 3.2.2 A comparative analysis of pharmacoeconomic guidelines in Europe regarding surrogate endpoints

In this study, we reviewed pharmacoeconomic guidelines from various European countries, limited to documents available in English. Among the countries analyzed, only three provided explicit definitions of surrogate endpoints. Portugal (Vinhas et al., 2021) defines surrogate endpoints exclusively as biomarkers. In contrast, France (Haute Autorité de Santé, 2020) and Ireland (Health Information and Quality Authority, 2018) classify them as intermediate endpoints. Ireland requires these endpoints to be objectively measurable, meaning that the outcomes must be quantified directly through standardized instruments or tests and should produce values that are independent of assessor interpretation. France, on the other hand, also accepts observational measures, meaning that outcomes can be based on systematic clinician or researcher observation of patient signs, symptoms, or behaviors, where structured assessments are applied but some degree of subjective judgment remains unavoidable.

As previously noted, the validation and transferability of surrogate endpoints are particularly important in the context of ATMPs. Portugal and Germany (Institut für Qualität und Wirtschaftlichkeit im Gesundheitswesen, 2023) are the only countries that explicitly state that transferability should be assessed based on the specific disease, population, and technology. However, the Portuguese guidelines make an exception for novel health technologies: in such cases, surrogate endpoints may be based on evidence from drugs with different mechanisms of action, and are considered acceptable only when no therapeutic alternatives exist or when treating serious conditions. England and Wales (National Institute for Health and Care Excellence, 2022), indicate that transferability should be assessed with respect to both the technology and the disease context, while France limits this consideration to disease specificity alone. Other countries do not explicitly address the transferability of surrogate endpoints.

Most countries (see Table 3) emphasize the need for a demonstrated causal relationship between the surrogate and clinical endpoint. Three countries refer to a staged validation process. Germany, for instance, only accepts validation based on meta-analyses, whereas alternative methods may be considered by other countries but require strong justification. Portugal is the only country to specify quantitative thresholds, requiring a correlation coefficient of 0.85 or 0.955 between the surrogate and clinical endpoint. Lastly, Scotland (Healthcare Improvement Scotland, 2022) and England and Wales note that validation requirements may vary in the context of orphan or rare diseases.

**TABLE 3:** HTA guidance on the use and preference for surrogate endpoints.

Country	Definition	Validity	Transferability
Germany	/	Validation requires staged validation, preferably meta-analysis, alternatives are only considered in justified exceptions. If a surrogate endpoint cannot be validated conclusively, the surrogate threshold effect concept can be used	Transferability is population-specific, technology-specific and disease specific
Norway	/	Validation requires a causal correlation	/
France	A biomarker or intermediate outcome that can be considered a relevant substitute for a clinical endpoint	Validation requires a causal correlation	/
Denmark	/	Validation requires a causal correlation	/
Ireland	An objectively measured endpoint expected to predict clinical benefit or harm, based on epidemiologic, pathophysiologic, therapeutic, or other scientific evidence	Validation requires a causal correlation and a comparable effect size	/
Austria	/	Validation requires a causal correlation	/
Portugal	A biomarker intended to replace a clinical outcome measure	Validation requires staged validation. Correlation coefficient values between 0.85 and 0.955 are given. If there is not a high correlation, the surrogate threshold effect can still be used	Transferability is population-specific, technology-specific and disease specific. To validate a surrogate endpoint for a new technology, use evidence from other drugs for the same indication only if no alternatives exist or added benefit is expected
England and Wales	/	Validation requires staged validation. For some rare diseases evidence levels may be lower	Transferability is population-specific and technology-specific
Scotland	/	Validation requires a causal correlation	/

### 3.3 Innovative trial design

Innovative clinical trial designs are increasingly recognized as essential for addressing key challenges in ATMP evidence generation, particularly the frequent reliance on single-arm studies and surrogate endpoints. These designs can maximize limited patient populations, integrate real-world data, and support earlier assessment of clinical outcomes. Below, we outline several innovative trial types and illustrate their potential contributions to ATMP development.

An adaptive trial allows pre-planned modifications based on interim data, such as adjusting sample size, modifying randomization ratios, adding or dropping treatment arms, or stopping early for success or futility (Bhatt and Mehta, 2016). This methodology is used in different innovative trial designs mentioned below.

A master protocol tests multiple treatments and/or subpopulations within a single overarching protocol, eliminating the need for separate studies. There are three main types: umbrella trials, which test multiple treatments for a single disease; basket trials, which test a single treatment across multiple diseases or subtypes; and platform trials, which allow multiple treatments to be evaluated adaptively over time (Woodcock and LaVange, 2017). This design is particularly useful for ATMPs because it enables efficient evaluation in small and heterogeneous populations, allows simultaneous testing of multiple strategies, and adapts to emerging data. For example, the GLO-BNHL study is a global, adaptive platform trial evaluating several novel agents, including CAR-T therapies, for pediatric and young adult patients with relapsed or refractory B-cell Non-Hodgkin lymphoma, allowing ineffective treatments to be dropped while promising ones continue.

Seamless trials combine multiple phases within a single protocol, often reusing patients and infrastructure. Inferentially seamless designs incorporate early-phase data into later analyses, increasing efficiency but requiring complex statistical planning, whereas operationally seamless designs maintain continuity across phases but rely only on confirmatory-stage data for final evaluation (Cuffe et al., 2014). The UX701 trial for Wilson disease, an operationally seamless Phase I/II/III study, exemplifies how this approach can accelerate development in rare diseases with recruitment challenges.

The N-of-1 trial, also known as a single-patient trial or personalized trial, is a research design where a single individual is the only participant and serves as their own control. In this design a patient receives an intervention alternating with a placebo or a different intervention, over a series of pre-defined periods (Samuel et al., 2023). The order of interventions is typically randomised and blinded to both the patient and the clinician to minimize bias. These trials are ideal for chronic stable health conditions. For a single-administration therapy like ATMPs, the traditional alternating N-of-1 design can be used. Since a classic crossover isn't feasible, the trial establishes a patient’s baseline before administering the therapy, followed by long-term monitoring of their efficacy and safety (Kim-McManus et al., 2024). This innovative design has been used with Milasen®, a patient-customized oligonucleotide therapy for Batten’s disease which demonstrates similarities to ATMPs despite not being strictly classified as one (Kim et al., 2019).

Pragmatic clinical trials are designed to evaluate the effectiveness of interventions under real-world conditions. These trials often rely on a broad range of outcomes derived from real-world data (RWD) (Ford and Norrie, 2016). For ATMPs, which frequently enter the market with limited clinical trial data, post-marketing RWE is particularly valuable in addressing evidence gaps and complementing initial trial findings by providing crucial long-term safety and effectiveness data from diverse patient groups in real-world settings. In the case of Strimvelis®, an analysis of treated patients, including those in the post-marketing phase, confirmed durable efficacy and a favorable risk–benefit profile (Migliavacca et al., 2024).

Innovative trial designs provide practical solutions to the limitations of single-arm studies and surrogate endpoints in ATMP development. Master protocols can integrate multiple sub-studies and shared control data, reducing reliance on independent comparators. Seamless and adaptive trials allow iterative modification of endpoints and sample sizes, supporting early evaluation and refinement of surrogate endpoints. Pragmatic trials leverage real-world data to provide context for comparative effectiveness when conventional randomization is not feasible. By combining these approaches, developers can generate more robust, interpretable evidence, contextualize surrogate endpoints, and strengthen the overall reliability of clinical and economic assessments for ATMPs.

## 4 Discussion

ATMPs are transforming the landscape of medical treatment by offering curative potential for previously intractable diseases. In this article, we reviewed the literature and analyzed European pharmacoeconomic guidelines to examine how key methodological clinical challenges for ATMPs such as the absence of appropriate comparators and the uncertainty of long-term clinical outcomes can be addressed in the context of reimbursement evaluation.

Most ATMPs gain market access based on single-arm studies (20/27), which frequently rely on surrogate endpoints (19/27). There is a clear need for stronger, more explicit guidance on assessing the feasibility of conducting an RCT, particularly when robust comparative effectiveness evidence is required. Currently, no standardized criteria exist, and feasibility evaluations are not consistently performed in collaboration with stakeholders at an early stage. When an RCT is deemed infeasible, there should be a well-supported rationale explaining why, including why innovative trial designs cannot address the identified challenges. In the absence of randomization, comparative evidence often relies on indirect ITCs, typically unanchored PAICs due to single-arm trials. However, without a randomised comparator, confounding factors may remain unevenly distributed, introducing bias. Recent research has shown considerable inconsistency in reporting the rationale for selecting effect modifiers and how balance between treatment populations was achieved. Ensuring methodological rigor and transparency in these steps is crucial to provide a reliable picture of comparative effectiveness. Pharmacoeconomic guidelines generally prioritize RCT evidence and accept ITCs only when direct comparisons are not feasible, yet they offer little clarity on when ITCs are appropriate. Perceptions of population-adjusted methods such as MAIC and STC also vary widely: many guidelines remain cautious due to the significant uncertainty these methods entail, while others allow greater flexibility if methodological choices are justified and potential biases transparently addressed.

The potentially curative nature of ATMPs poses significant challenges to traditional HTA frameworks, often necessitating reliance on surrogate endpoints in the absence of long-term clinical data. The validation of such endpoints typically depends on evidence from RCTs, and their applicability is highly context-specific. While rigorous validation remains essential, current methodological frameworks are frequently insufficient to address the unique requirements of ATMPs. Moreover, most pharmacoeconomic guidelines do not provide explicit criteria for determining acceptable levels of surrogate endpoint validation or for managing situations in which RCT-based validation is infeasible and transferability conditions are not met. This uncertainty is further compounded by the difficulty of extrapolating short-term surrogate endpoints, outcomes should be left out to long-term clinical and economic impacts.

Recent regulatory developments illustrate alternative approaches to evidentiary uncertainty. The FDA’s *Plausible Mechanism Pathway* allows early clinical evidence to be considered when supported by a well-substantiated biological rationale, even when conventional surrogate endpoint validation through randomised trials is not feasible (Prasad and Makary, 2025). While developed in a regulatory context, this approach highlights considerations that are also relevant for HTA of ATMPs, particularly in settings characterized by small populations, single-arm studies, and immature outcome data, and underscores the importance of transparency and post-marketing evidence generation. To mitigate these challenges, innovative trial designs offer a structured approach to improving evidence generation. Master protocols, seamless trials, and pragmatic designs can maximize the information obtained from limited patient populations, incorporate real-world evidence, and support iterative evaluation of surrogate endpoints. While they cannot replace randomised comparators, these approaches can enhance the interpretability and contextual relevance of single-arm studies. Formally incorporating such designs into HTA frameworks and outcomes-based MEAs could help reduce uncertainty in reimbursement decisions, even in the absence of conventional RCTs.

This lack of standardization risks delaying patient access to potentially transformative treatments. To mitigate this, early and structured dialogue between developers and payers is essential to reduce evidentiary uncertainty and streamline the reimbursement process. An example of such collaborative efforts is the newly established Joint Clinical Assessment (JCA) framework under the EU HTA Regulation (Regulation EU, 2021). Building on the groundwork laid by EUnetHTA, the JCA provides a formal mechanism for cross-country cooperation through joint scientific consultations, aiming to ensure more consistent, transparent, and efficient HTAs across Europe. Possibly supported by bodies such as the JCA, it will become increasingly important for HTA agencies to establish clear guidance on the use of PAICs and surrogate endpoints. New ATMPs will continue to face challenges related to the lack of suitable comparators and the absence of long-term clinical data. It is therefore crucial that HTA agencies acknowledge these limitations and provide explicit criteria for when ITCs are appropriate, as well as for the acceptable use and extrapolation of surrogate endpoints.

Given the frequent immaturity of clinical data at market entry, post-marketing evidence generation will continue to play a pivotal role. Here, RWE becomes indispensable (Dang, 2023). Initiatives like the European Health Data Space have the potential to facilitate robust RWE collection across jurisdictions, provided that challenges related to data interoperability, quality, and governance are adequately addressed (Regulation EU, 2025).

Finally, Managed Entry Agreements (MEAs) will likely remain a key tool for managing uncertainty (Vreman et al., 2020). Outcomes-based MEAs, which link reimbursement to real-world effectiveness, are particularly relevant (Greco et al., 2025). However, their feasibility is limited by the same challenges that affect initial evidence generation, including single-arm trials, lack of comparators, and reliance on surrogate endpoints. Without robust comparators and validated endpoints, it is difficult to attribute observed outcomes to the therapy and to predict long-term benefit. Successful implementation also requires strong real-world evidence infrastructure, including standardized data collection, transparent registries, and consensus on meaningful endpoints. Without these elements, operational complexity may outweigh benefits, delaying access and threatening sustainable reimbursement (Dabbous et al., 2020).

It is important to acknowledge several limitations of this study. The literature was not identified through a systematic search and the synthesis was narrative in nature, which may have resulted in the omission of relevant guidelines. In addition, the selection of materials was performed by a single reviewer, potentially introducing selection bias. Furthermore, the inclusion was restricted to guidelines available in English, which may have limited the comprehensiveness and representativeness of the findings.

## 5 Conclusion

Our study has found that there is a need to further optimize methodologies to handle evidence from single-arm trials and to project long-term outcomes, both of which are common in ATMP development. Nonetheless, a certain level of clinical uncertainty will always be inherent in the reimbursement evaluation of these innovative therapies. This makes early and constructive dialogue between industry and payers essential. The task for modern healthcare systems is to manage these clinical uncertainties associated with ATMPs in a way that ensures their equitable and timely patient access without creating new barriers to care.
